# Replication dataset: the effect of compassion, biological sex, and gender identity threat on jerky choice

**DOI:** 10.1016/j.dib.2021.107595

**Published:** 2021-11-19

**Authors:** Attila Pohlmann

**Affiliations:** Universidad San Francisco de Quito USFQ, Ecuador

**Keywords:** Carnism, Gender identity threat, Masculinity, Psychic cost, Ethical consumption, Meat paradox, Moral self-concept, Compassion, Plant-based diet, Vegetarianism, Veganism, Meat attitudes

## Abstract

When it comes to food choices, high levels of trait compassion should decrease a person's likelihood to choose meat compared to a plant-based alternative [1], [2], [3], [4]. Because meat advertising often threatens masculinity, for men, this effect is expected to be moderated by gender identity threat. The data provided with this article were collected online from 1,350 participants to conduct a replication of study 1 in “The taste of compassion: Influencing meat attitudes with interhuman and interspecies moral appeals” [5]. The original study reports that men with high trait compassion [6] are significantly less likely to choose a vegetarian jerky–and more likely to choose a meat jerky instead–if masculinity is threatened. The replication is successful if the age range of participants between the two studies is matched. The size and direction of the effect tested in the replication study is comparable to that in the original study. This outcome suggests that the formation and the processing of meat attitudes depend on life stage, and it points to additional avenues for research in the fields of nutrition, social psychology, marketing, and consumer behavior. Additional variables in the dataset (e.g., items of the composite trait compassion variable, meat avoidance intent, social identity based on diet, and dietary pattern adherence [7], [8], [9], [10], [11]) may be used to develop and/or test hypotheses relating to meat attitudes and food-related choice behaviors. A print-out of the survey instrument, the dataset including scale items, and a script to perform the analysis are provided.

## Specifications Table

**Table:** 

Subject	Social and Personality Psychology
Specific subject area	Psychology of Meat Consumption
Type of data	Table
How data were acquired	Participants filled out an electronic survey using the Qualtrics platform. The survey print-out is provided in the data repository.
Data format	Raw
Parameters for data collection	Data are provided from all participants who started the survey.
Description of data collection	Data were collected online using Mechanical Turk.
Data source location	Participants indicated residence in the United States.
Data accessibility	Repository name: The Open Science Framework Data identification number: 10.17605/OSF.IO/NWFDG Direct URL to data: https://osf.io/nwfdg/
Related research article	Pohlmann, Attila “The taste of compassion: Influencing meat attitudes with interhuman and interspecies moral appeals.” *Appetite* (2021): 105654.

## Value of the Data

• Aside from providing a subsample to replicate the effect found in the original study [5], the entire dataset, collected from 1350 participants, provides additional information on psychological individual difference variables (e.g., trait compassion [6]), meat avoidance intent, social identity based on diet, and dietary pattern adherence [7], [8], [9], [10], [11] from a large sample of the US population. Combined with demographic information, these data can provide insight on dietary protein choices, which have implications for environmental, societal, and individual health.
• Investigators in the fields of nutrition, social psychology, marketing, and consumer behavior can benefit from analyzing these data further to establish and/or verify relationships between individual difference variables and food-related attitudes and behaviors. For instance, the results from the replication attempt suggest that cognitive processes, on which the formation of meat attitudes depends, are influenced by life stage (i.e., age).
• To develop and test additional hypotheses related to meat attitudes and choice behaviors, researchers can use the demographic information (e.g., sex, age, race) and the supplementary information contained in the scale items for the psychological trait measure, questionnaire items regarding diet, social identity based on diet, and dietary pattern adherence. Researchers can also benefit from the dataset to estimate effect sizes for replication studies and/or to conduct power analyses.

## 1 Data Description

The first study in “The taste of compassion: Influencing meat attitudes with interhuman and interspecies moral appeals” [5] tests the effect of compassion and gender identity threat on consumers’ choice between a meat jerky and a plant-based soy jerky. Due to the compassion-vegetarian link, people with higher levels of trait compassion are expected to choose the vegetarian soy jerky option more frequently compared to meat jerky. However, due to the influence of advertising on meat attitudes, this effect is expected to be moderated by gender identity threat, such that it increases the likelihood to choose the meat jerky for men and decreases the likelihood to choose the meat jerky for women. The original study was conducted in a laboratory setting with undergraduate students, but due to its sample size (*N* = 209), the probability of a Type II error is very high. To address this shortcoming, a replication study was conducted. A power analysis estimates that about 425 male participants are required to detect the effect of interest at alpha = .05 and beta = .80; see online appendix in [5]. Based on this analysis, the original survey instruments were adapted for an online survey; a replication attempt in a laboratory setting would have been impractical and costly. Participants were recruited on Amazon Mechanical Turk in April 2021. The target sample size was set at 1,200 participants. The final raw dataset contains responses from 1,350 participants.

The entire replication dataset can be downloaded as SPSS data (.sav) or as comma-separated values (.csv). A printout of the Qualtrics survey that was used to collect data, as well as the SPSS script to calculate combined variables and to perform the analysis are also available in the online data storage. The replication dataset contains the main experimental variables: gender identity threat (manipulated with false feedback on gender knowledge quiz), trait compassion (measured using the Santa Clara Brief Compassion Scale (SCBCS) [6]), biological sex, and, the outcome variable, choice between meat-based (beef, turkey) or plant-based jerky (soy). The names and descriptions of additional variables, scale items, as well as variable means (M) and standard deviations (SD) are provided in Table 1.

**Table 1:** List of variables and descriptive statistics for replication dataset.

Variable	Description	Scale	M	SD
BIOSEX	Biological Sex	0=female, 1=male	.47	.50
AGE18	Age in years	18-71	39.34	13.00
AGE_FILTER	Filter Variable, Age 18-28	0=not selected, 1=selected	.21	.41
SCBCS_1	Santa Clara Brief Compassion Scale, Item 1	When I hear about someone (a stranger) going through a difficult time, I feel a great deal of compassion. 1=not at all true of me, 5=very true of me	3.74	1.00
SCBCS_2	Santa Clara Brief Compassion Scale, Item 2	I tend to feel compassion for people, even though I do not know them.	3.73	1.00
SCBCS_3	Santa Clara Brief Compassion Scale, Item 3	One of the activities that provide me with the most meaning to my life is helping others in the world when they need help.	3.61	1.03
SCBCS_4	Santa Clara Brief Compassion Scale, Item 4	I would rather engage in actions that help others, even though they are strangers, than engage in actions that would help me.	3.35	1.05
SCBCS_5	Santa Clara Brief Compassion Scale, Item 5	I often have tender feelings toward people (strangers) when the seem to be in need.	3.69	1.01
SCBCSCTR	Santa Clara Brief Compassion Scale. Averaged, centered score	-2=low compassion, 2=high compassion	.62	.84
Q26-Q84	Gender Knowledge Quiz Items	Randomized, unscored Gender Knowledge Quiz	n/a	n/a
Q87, Q90, Q93, Q96	Gender Identity Threat Manipulation	False feedback on Gender Knowledge Quiz	n/a	n/a
GITHREAT	Gender Identity Threat Manipulation	1=Gender Identity Threat, 0=Non-Threat	.50	.50
MNPCHK_1	Manipulation Check, Item 1	Are you surprised by your score? 1=not at all surprised, 5=very surprised	3.21	1.18
MNPCHK_2	Manipulation Check, Item 2	Would other people who know you agree with your test score and characterize you accordingly? 1=Strongly disagree, 5=Strongly agree	3.29	.96
CHOOSEDV	Jerky Snack Choice	3=Beef 4=Turkey 5=Soy	3.83	.80
MEATJRKY	Meat Jerky (computed)	0=Soy 1=Meat	.75	.44
CATEG_MAI_1	Meat Avoidance Intent, Item 1	I avoid eating red meat. 0=no, 1=yes	.37	.48
CATEG_MAI_2	Meat Avoidance Intent, Item 2	I avoid eating meat: any animal flesh, e.g. beef, pork, seafood, chicken, etc.	.20	.40
CATEG_MAI_3	Meat Avoidance Intent, Item 3	I avoid eating any product that comes from an animal.	.15	.35
OMNIVEG_1	Dietary Identification, Item 1	I am an omnivore. 1 = Strongly disagree, 10 = Strongly agree	7.31	3.01
OMNIVEG_2	Dietary Identification, Item 2:	I am a vegetarian.	3.31	3.05
OMNIVEG_3	Dietary Identification, Item 3	I am a vegan.	2.74	2.78
DPA_1	Dietary Pattern Adherence, Item 1	I always adhere to these dietary statements. 1 = Strongly disagree, 10 = Strongly agree	6.76	2.50
DPA_2	Dietary Pattern Adherence, Item 2	I occasionally make exceptions.	5.80	2.77
RSTRDIET	Restrictive Diet	Are you currently on a calorie-restrictive diet? 0=no, 1=yes	.25	.44
ETHNICITY_1	Participant Race, Item 1	1=White	.67	.47
ETHNICITY_2	Participant Race, Item 2	1=Black or African American	.11	.31
ETHNICITY_3	Participant Race, Item 3	1=American Indian or Alaska Native	.01	.12
ETHNICITY_4	Participant Race, Item 4	1=Asian	.10	.30
ETHNICITY_5	Participant Race, Item 5	1=Native Hawaiian or Pacific Islander	.00	.06
ETHNICITY_6	Participant Race, Item 6	1=Other	.02	.13
ETHNICITY_6_TEXT	Participant Race, Item 6: Other, form field entry	String	n/a	n/a

## 2 Experimental Design, Materials and Methods

The replication study was preregistered with The Open Science Framework (https://osf.io/fs3md). A successful replication was defined as “significant three-way interaction and/or at least the contrast of interest: masculinity threat increases the likelihood of men with high trait compassion to choose the meat jerky rather than the plant-based jerky. Possibly, age, ethnic background, and self-reported dietary preferences/priorities/pattern adherence can serve as covariates to reduce error variance and/or to match samples.” The original study was conducted in a laboratory setting with undergraduate students whereas the replication study was conducted entirely online with a random sample of participants recruited via Amazon Mechanical Turk. This circumstance has implications for the location, procedure, and population of the replication study. The stimuli were the same as in the original study.

Participants provided demographic information, completed the SCBCS [6], and were randomly assigned to the gender identity threat and non-threat conditions. Gender identity threat was manipulated with false feedback on a gender knowledge quiz. The masculinity threat condition showed a graph with the ostensible quiz results indicating that a participant's performance was in the lower 27th percentile of male peer performance. In the non-threat condition, the graph indicated performance in the upper 73rd percentile. The false feedback was reversed for women, so that a high score on masculine knowledge items represented the femininity threat condition. All quiz items and the false results pages are reproduced in the survey printout, available in the online appendix. Then, participants were asked to review ingredients and nutritional information for jerky snacks presented in three standard FDA tables, one of which they could subsequently choose. The three options (beef, turkey, soy) were ostensibly identical in terms of calories (70 kcal), carbohydrates (5 g), fat (0 g), and protein (12 g). Finally, potential control variables pertaining to meat avoidance intent were captured (e.g., I avoid eating meat, I avoid eating any product that comes from an animal [8]), social identity based on diet (e.g., I am an omnivore, I am a vegetarian [2,10]), dietary pattern adherence (e.g., I always adhere to these dietary statements, I occasionally make exceptions [7]), and calorie-restrictive diets (e.g., Atkins, calorie-counting, fasting, WeightWatchers). At the end of the survey, participants provided information on their race.

The five items of the Santa Clara Brief Compassion Scale [6] (5 items, Cronbach's Alpha = .88), measured on a 5-point scale, were averaged and centered on zero (SCBCSCTR = 3 - MEAN (SCBCS_1, SCBCS_2, SCBCS_3, SCBCS_4, SCBCS_5)). To test whether the replication was successful, in a first step, using Hayes’ Process macro configured for model 13 (https://www.processmacro.org, [12]), the same statistical analysis with identical variable coding as in the original study was performed: a 2 (trait compassion: high = 90th percentile vs. low = 10th percentile) x 2 (biological sex: female = 0 vs. male = 1) x 2 (gender identity threat = 1 vs. non-threat = 0) between subjects design with jerky choice (meat = 1, soy = 0) as outcome. The logistic regression analysis included 1240 participants (670 female) and yielded a non-significant three-way interaction term (B = -.14, SE = .34, 95% CI -.79 to .52, N.S.); individual contrasts were not statistically significant either.

In a second step, the age range of the participants in the replication study was matched to that of the sample in the original study (M = 22.37 years, SD = 4.97, see [5]). The gender identity threat manipulation check was successful; participants in the threat condition indicated less agreement with the statement that others would characterize them according to the mock score on the gender knowledge quiz (MNPCHK_2, M_threat_ =3.09, SD = 1.05, M_non-threat_ = 3.44, SD = .87, *F*(_1,260_ = 8.59, *p* < .01). A 10-point covariate (DPA_2) to reduce error variance in dietary pattern adherence [7] was included in the model (“I occasionally make exceptions [to previously stated dietary preferences, piped into the survey question]” 1 = Strongly disagree, 10 = Strongly agree, M= 5.80, SD = 2.78).

If the age range of the sample in the replication study is constrained to 18–28 years (AGE_FILTER), the tested effect of the replication study is statistically significant and in the expected direction: the planned contrast with the constrained sample (*N* = 261, 153 female) shows that masculinity threat increases highly compassionate men's (SCBCSCTR, 90th percentile = 1.60) likelihood to choose the meat jerky compared to the non-threatened male group (p_threat_ = .88 vs. p_non-threat_ = .67, B = 1.29, SE = .78, one-tailed 95% CI .01 to 2.57). The size of the replicated effect is comparable to the one detected in the original study (p_threat_ = .92 vs. p_non-threat_ = .59), although the difference between probabilities in the replication study is smaller compared to the original study (Δp_replication_ = .21 vs. Δp_original_ = .33).

By the criteria set out in the replication study registration, the replication attempt can be deemed successful and yields insight for future research. There is a caveat however; by constraining the age range in the replication study, the sample size is decreased. Potential concerns should be attenuated by the a-priori power analysis and the additional replication of the effects of interest in the associated article [5]. Considering the finding that the replication is only successful within a specific age range of young adults, age–or life stage–appears to play an important role in how meat-related messages are absorbed and processed, and how they ultimately influence consumer-relevant behavior.

## Ethics Statement

All procedures involving human participants were performed in accordance with the ethical standards of the Institutional Review Board of the University of Hawaiʻi at Mānoa (CHS#21360) and the Comité de Éticade Investigación en Seres Humanos Universidad San Francisco de Quito (2019-034IN). Informed consent was obtained from participants, and they were free to withdraw at any time. Participants were debriefed at the end of the survey.

## CRediT Author Statement

**Attila Pohlmann:** Conceptualization, Methodology, Data curation, Formal analysis, Visualization, Writing – review & editing.

## Declaration of Competing Interest

The author declares that he has no known competing financial interests or personal relationships which have or could be perceived to have influenced the work reported in this article.

## Appendix Supplementary materials

Image, application 1

Image, application 2

Image, application 3
